# The European Medicines Agency Review of Crizanlizumab for the Prevention of Recurrent Vaso-Occlusive Crises in Patients With Sickle Cell Disease

**DOI:** 10.1097/HS9.0000000000000604

**Published:** 2021-06-28

**Authors:** Julio Delgado, Caroline Voltz, Milena Stain, Tuomo Lapveteläinen, Susanne Urach, Johanna Lähteenvuo, Karri Penttilä, Christian Gisselbrecht, Harald Enzmann, Francesco Pignatti

**Affiliations:** 1 Oncology & Haematology Office, European Medicines Agency, Amsterdam, The Netherlands; 2 Department of Haematology, Hospital Clinic, Barcelona, Spain; 3 Bundesamt fur Sicherheit im Gesundheitswesen, Vienna, Austria; 4 Committe for Medicinal Products for Human Use, European Medicines Agency, Amsterdam, The Netherlands; 5 Lääkealan turvallisuus- ja kehittämiskeskus, Fimea, Finland; 6 Hopital Saint Louis, Paris, France; 7 Bundesinstitut für Arzneimittel und Medizinprodukte, Bonn, Germany

## Abstract

Crizanlizumab is a monoclonal antibody that binds to P-selectin. On October 28, 2020, a conditional marketing authorization valid through the European Union (EU) was issued for crizanlizumab for the prevention of recurrent vaso-occlusive crises (VOCs) in patients with sickle cell disease aged 16 years or older. Crizanlizumab was evaluated in a phase 2, double-blind, placebo-controlled randomized multicenter trial comparing high-dose (5 mg/kg) crizanlizumab, low-dose (2.5 mg/kg) crizanlizumab and placebo in patients with a history of 2–10 VOCs in the previous year. Patients who were receiving concomitant hydroxycarbamide (HC) as well as those not receiving HC were included in the study. The primary endpoint of the trial was the annual rate of sickle cell-related pain crises as adjudicated by a central review committee. High-dose crizanlizumab led to a 45.3% lower median annual rate of sickle cell-related pain crises compared to placebo (*P* = 0.010), with no statistically significant difference for the low dose. Treatment with high-dose crizanlizumab led to similar incidences of adverse events (AEs), grade 3 AEs, and serious AEs compared to placebo. Most frequently observed AEs that occurred more often in the crizanlizumab arm compared to placebo were infusion related reactions (34.8% versus 21%), arthralgia (18.2% versus 8.1%), diarrhea (10.6% versus 3.2%), and nausea (18.2% versus 11.3%). The aim of this article is to summarize the scientific review of the application leading to regulatory approval in the EU.

## Introduction

Sickle cell disease (SCD) is a heritable disease mainly prevalent in sub-Saharan Africa, India and the Middle East, but with a steady increase in the European Union due to migration.^1–5^ SCD is the result of a point mutation in the beta globin gene, leading to deformed red blood cells (RBCs), anemia, hemolysis, painful vaso-occlusive crises (VOC), permanent organ damage, and reduced life expectancy.^6,7^ The management of SCD mainly includes the treatment of clinical manifestations and preventive measures. The treatment of VOC includes hydration, anti-inflammatory agents, painkillers, and antibiotics in the case of fever or suspected infection. Life-threatening or severe complications, such as acute chest syndrome (ACS) or stroke, often require RBC transfusions or RBC exchange.^8^ Hydroxycarbamide (HC) can decrease the frequency and severity of VOC and reduce the transfusion burden, but not all eligible patients can tolerate it, and some still experience VOC despite taking the drug.^9–11^ The only curative option for SCD is allogeneic hematopoietic cell transplantation (alloHCT), which is generally reserved for children or adolescents with a matched sibling donor.^12^

On May 29, 2019, Novartis Europharm Limited applied for a marketing authorization via the European Medicines Agency (EMA) centralized procedure for crizanlizumab (trade name Adakveo). Crizanlizumab had been designated orphan medicine by the European Commission (EC) on August 9, 2012 for the treatment of SCD. To qualify for orphan designation, a medicine must be intended for the treatment, prevention. or diagnosis of a life-threatening or chronically debilitating disease, the prevalence of the condition in the European Union (EU) must not be >5 in 10,000, and the medicine must be of significant benefit to those affected by the condition. Benefits of an orphan designation include a 10-year market exclusivity and access to scientific advice, which the marketing authorization holder (MAH) received on April 26, 2018.

The review of the benefit–risk balance was conducted by the Committee for Medicinal Products for Human Use (CHMP), and the positive opinion was issued on July 23, 2020. The indication approved in the EU is as follows: “Adakveo is indicated for the prevention of recurrent VOCs in sickle cell disease patients aged 16 years and older. It can be given as an add-on therapy to hydroxyurea (HU)/HC or as monotherapy in patients for whom HU/HC is inappropriate or inadequate.” The aim of this article is to summarize the scientific review of the application leading to the regulatory approval of crizanlizumab in the EU.

## Nonclinical aspects and clinical pharmacology

Crizanlizumab is a monoclonal antibody (mAb) that binds to P-selectin, thus blocking its interaction with its ligands. P-selectin is an adhesion molecule expressed on activated vascular endothelial cells and platelets and is a key molecule involved in leukocyte extravasation upon inflammation. It is expressed at high levels in patients with SCD and is considered a key factor in the pathogenesis of VOC.^13,14^

The pharmacokinetics (PK) of crizanlizumab were characterized in healthy subjects and patients with SCD in 4 clinical studies (A2101, A2201, A2102, and A2202) evaluating doses ranging from 0.2 to 8 mg/kg. The main pharmacodynamic (PD) marker was the percentage of ex vivo P-selection inhibition measured by a competitive binding assay that mimics the in vivo mechanism.

A clear correlation was observed between the dose and the PD effect. Considering the target population of patients with SCD, the mean trough percentage of P-selectin inhibition 4 weeks postdose was 24%–40% (2.5 mg/kg), 63.5%–76.3% (5 mg/kg), and 2.8%–6.05% (placebo). Additional PK data will be generated in the ongoing phase III trial (A2301).

Crizanlizumab was first developed by Reprixys Pharmaceuticals and acquired by Novartis in 2016. Clinical studies A2101 and A2201 used the Reprixys-manufactured crizanlizumab (SelG1), whereas the ongoing A2202 and A2301 studies are using the Novartis-manufactured crizanlizumab (SEG101).

## Trial design

The submission was based on a single placebo-controlled, double-blind, randomized phase II study (A2201) evaluating the efficacy and safety of crizanlizumab in the prevention of VOCs in patients with SCD.^15^ In this trial, patients with SCD from 16 to 65 years of age were randomized in a 1:1:1 ratio to 5 mg/kg (high dose [HD]) crizanlizumab, 2.5 mg/kg (low dose [LD]) crizanlizumab or placebo. Patients were stratified by concomitant HC use (yes/no) and the number of VOCs in the preceding 12 months (2–4 versus 5–10 crises). Patients on a chronic transfusion program, receiving chronic anticoagulation, or with a history of stroke within the past 2 years were not included. The drug (or placebo) was infused over 30 minutes by intravenous infusion at day 1, day 15, and then every 4 weeks through week 50, for a maximum of 14 doses.

The primary endpoint was the annual rate of sickle cell-related pain crises (SCPCs), which had to meet all the following criteria: (1) acute episode of pain; (2) no known cause for pain other than a VOC; (3) requirement of a visit to a medical facility; and (4) requirement of oral/parenteral opioids or parenteral anti-inflammatory agents. ACS, hepatic sequestration, splenic sequestration, and priapism were also considered SCPC. All SCPCs were independently adjudicated by a central review committee (CRC). In the case of early dropouts or patients lost to follow up, the annual rate of SCPC was calculated by extrapolation to 1 year. A stratified Wilcoxon rank-sum test was used for the analysis of the primary endpoint. Median differences and 95% confidence intervals (CIs) for the median difference were estimated using the Hodges–Lehmann (HL) method.

The key secondary endpoint was the annualized rate of days hospitalized. Other secondary endpoints were time to first and second SCPC, annual rate of uncomplicated SCPC (ie, excluding ACS, hepatitis sequestration, splenic sequestration, and priapism), and annual rate of ACS and patient reported outcomes.

A sample size of 50 patients per arm was planned, having a 90% power to detect a 40% reduction in SCPC. Assuming a 15% dropout rate, approximately 174 patients would be required in total. The primary endpoint and the key secondary endpoint were controlled for type I error using hierarchical testing (ie, the primary endpoint served as a gatekeeper for the key secondary endpoint).

## Clinical efficacy

A total of 198 subjects were randomized: 67 to HD crizanlizumab, 66 to LD crizanlizumab, and 65 to placebo (intention-to-treat [ITT] population). Six (3%) of these patients did not receive study drug: 1 in the HD crizanlizumab arm, 2 in the LD crizanlizumab arm, and 3 in the placebo arm (modified ITT [mITT] population). The per protocol (PP) population comprised 125 (63.1%) patients who received at least 12 of the 14 planned doses, completed a visit 14 days after the last dose and had no other major protocol violations. Sixty-nine (34.8%) patients discontinued from the study, with no imbalances among treatment arms regarding the number of patients with early discontinuation or the reason for early discontinuation. Patients’ baseline characteristics were well balanced across treatment arms. The proportion of patients on concomitant HC therapy among those allocated to HD crizanlizumab, LD crizanlizumab, and placebo was 64.2%, 60.6%, and 61.5%, respectively. Moreover, 37.3%, 37.9%, and 36.9% of patients allocated to the same arms had 5–10 VOCs in the previous 12 months.

The median annual rate of SCPC was 1.63 versus 2.98 for patients receiving HD crizanlizumab versus placebo, respectively (45.3% reduction in the HD crizanlizumab arm, *P* = 0.010). LD crizanlizumab led to a 32.6% reduction in the median annual rate of SCPC compared to placebo (2.01 versus 2.98, *P* = 0.18) (Table 1). Sensitivity analyses were performed in the mITT and PP patient populations and were concordant with these findings.

**Table 1.** Key Favorable and Unfavorable Results of Crizanlizumab for Patients Aged 16 Years and Over (A2201 Study, Cutoff Date: October 19, 2018)

Effect	Unit	High-dose crizanlizumab (n = 67)	Low-dose crizanlizumab (n = 66)	Placebo (n = 65)	Uncertainties, strength of evidence
**Favorable effects**					
The initial submission of the efficacy analyses was performed on CRC-adjudicated data using the HL estimate as well as simple annualization for imputation of missing data					
Annual rate of SCPC	Standard median HL median	1.63 2.00	2.01 2.50	2.98 3.49	Uncertainties regarding statistical methods and concomitant use of HC
Annual rate of days hospitalized	Standard median HL median	4.00 12.48	6.87 9.01	6.87 13.00	
Annual rate of uncomplicated SCPC	Standard median HL median	1.08 1.97	2.00 2.01	2.91 3.00	
Time to first SCPC	Median (mo)	4.07	2.20	1.38	
Time to second SCPC	Median (mo)	10.32	9.20	5.09	
Reanalysis requested by the CHMP, using investigator-adjudicated, negative binomial regression and “jump to reference” imputation method					
Annual rate of SCPC	Mean (±SD) RR (95% CI)	3.62 (4.12) 0.74 (0.52–1.06)		4.95 (5.29)	
Annual rate of days hospitalized	Mean (±SD) RR (95% CI)	17.31 (32.94) 0.72 (0.36–1.45)		24.41 (43.37)	
Annual rate of uncomplicated SCPC	Mean (±SD) RR (95% CI)	3.39 (3.99) 0.72 (0.49–1.05)		4.79 (5.49)	
Time to first and second SCPC	Months HR (95% CI)	3.78 0.54 (0.36–0.81)		1.15	
**Unfavorable effects**					
Drug-related AEs	Patients (%)	27 (40.9) 10 (22.2)^*a*^	21 (32.8)	15 (24.2)	
Drug-related SAEs	Patients (%)	6 (9.1) 0 (0)^*a*^	5 (7.8)	2 (3.2)	
Infusion-related reactions	Patients (%)	23 (34.8) 14 (31.1)^*a*^	20 (31.3)	13 (21)	
Effects on hemostasis or hemorrhage	Patients (%)	11 (16.7) 5 (11.1)^*a*^	7 (10.9)	8 (12.9)	
Infections	Patients (%)	35 (53) 20 (44.4)	(45.3)	33 (53.2)	

AEs = adverse events; CHMP = Committee for Medicinal Products for Human Use; CI = confidence interval; CRC = central review committee; HC = hydroxycarbamide; HL = Hodges–Lehmann; SAEs = severe adverse events; SCPC = sickle cell-related painful crisis; SD = standard deviation.

^*a*^A2202 study (n = 45).

The median annualized rate of days hospitalized was 4.0 versus 6.87 days for patients receiving HD crizanlizumab versus placebo (*P* = 0.45); and 6.87 versus 6.87 for patients receiving LD crizanlizumab versus placebo (*P* = 0.837).

The median time from randomization to first SCPC was 4.07 versus 2.2 versus 1.38 months for the HD crizanlizumab versus LD crizanlizumab versus placebo arms, respectively. The median time to second SCPC was 10.32 versus 9.20 versus 5.09 months for the HD crizanlizumab versus LD crizanlizumab versus placebo groups, respectively.

Median annual rates of uncomplicated SCPC were 1.08 versus 2.91 for patients receiving HD crizanlizumab versus placebo, respectively (62.9% reduction), while LD crizanlizumab led to a 31.3% reduction in the median annual rate of uncomplicated SCPC compared to placebo (2.00 versus 2.91). Due to the rare occurrence of ACS, no treatment differences were observed across treatment groups.

## Clinical safety

The safety database comprises 66 patients receiving HD crizanlizumab (SelG1), 64 patients receiving LD crizanlizumab (SelG1), 62 patients receiving placebo (all 3 groups from study A2201), and 45 patients receiving crizanlizumab (SEG101) from study A2202. Safety data from the ongoing A2202 study (an open label study in patients with SCD to assess PK, PD, and safety of the new formulation SEG101) was updated during the evaluation (last cutoff date: October 4, 2019).

In the A2201 study, patients were exposed to crizanlizumab (5.0 versus 2.5 mg/kg) for a mean of 43.8 versus 46.3 weeks, respectively, compared to placebo (43.7 wks). In the A2202 study, the mean exposure was 64.7 weeks at the last update.

The incidence of adverse events (AEs) in the A2201 study was 86.4%, 87.5%, and 88.7% for the HD crizanlizumab, LD crizanlizumab, and placebo groups, respectively, and 93.3% in the A2202 study. Grade ≥3 AEs were observed in 18.2%, 20.3%, and 19.4% of patients receiving HD crizanlizumab, LD crizanlizumab, and placebo, respectively (A2201 study), but were higher (40.0%) in patients receiving crizanlizumab in the A2202 study. Serious AEs (SAEs) were documented in 25.8%, 31.3%, and 27.4% of patients given HD crizanlizumab, LD crizanlizumab, and placebo, respectively (A2201 study) and 0% of patients enrolled in the A2202 study.

AEs whose incidence was ≥5% greater in the HD crizanlizumab arm compared to placebo included arthralgia (18.2% versus 8.1%), diarrhea (10.6% versus 3.2%), and nausea (18.2% versus 11.3%).

Regarding AEs of special interest (AESI), representing groupings of different MedDRA preferred terms, infections occurred in 53.0% (all grades) and 7.5% (grade ≥ 3) of patients receiving HD crizanlizumab, compared to 53.2% and 4.8% of patients receiving placebo; signs and symptoms of potential infusion-related reactions (IRRs) occurred in 34.8% of patients from the HD crizanlizumab arm compared to 21% of patients from the placebo arm, with no grade ≥3 IRRs in either arm; and effects on hemostasis or hemorrhage were observed in 16.7% of patients receiving HD crizanlizumab compared to 12.9% of patients receiving placebo. Drug-induced liver injury was not observed in any patient.

## Benefit–risk assessment

The current treatment of SCD is mostly aimed at its clinical manifestations (pain episodes, infections, ACS, or stroke). The only curative option is alloHCT, which is only available for selected patients with matched sibling donors. HC is approved in the EU for the prevention of SCPCs in adults, adolescents and children older than 2 years, but it is not always well tolerated and may yield insufficient responses. Crizanlizumab addresses an unmet need by providing an alternative treatment option besides HC and by reducing the requirement for on-demand symptomatic control.

The evidence of efficacy comes from a phase II, double-blind, placebo-controlled randomized study. The primary endpoint used in this trial (annual rate of SCPCs) was considered relevant since SCPCs represent a substantial burden for patients and can result in significant organ failure and mortality. Although the results of the prespecified primary and secondary analyses showed favorable effects for crizanlizumab at the 5-mg/kg dose, the magnitude of this benefit was difficult to assess due to uncertainties of the statistical methodology. First, a good clinical practice (GCP) inspection revealed that CRC-adjudicated data were not sufficiently robust, whereas investigator-adjudicated results were more reliable. Second, even though the difference between treatment arms was significant using a stratified Wilcoxon rank-sum test, the CI of the difference between the HL medians of the annual rate of SCPCs included 0. This duality between test and estimation method was questioned. Third, more than one third of randomized patients prematurely discontinued from the study, and therefore, the handling of missing values had a strong impact on the treatment effect estimates. The imputation using simple annualization was questioned, as was the assumption that the SCPC would remain constant after discontinuation. Still, the reanalysis provided by the MAH following recommendations by the CHMP confirmed the beneficial effect of crizanlizumab over placebo. This reanalysis was based on investigator-adjudicated SCPCs, a negative binomial regression instead of the HL method, and additional imputation methods: (1) by pain crises data before randomization and (2) reference-based (“jump-to-reference”) multiple imputation (Table 1).

Although the pivotal study was not powered to demonstrate differences between subgroups, these analyses consistently suggested a therapeutic benefit across all subpopulations, including patients with and without concomitant HC therapy. Clinical data using the SEG101 formulation, planned for commercial use, was sparse, with only preliminary PK/PD/safety results available from the ongoing A2202 study. After a thorough analysis of these data, comparability between SelG1 and SEG101 was adequately established.

Crizanlizumab is a first-in-class mAb that binds to P-selectin, thus reducing cell adhesion and the risk of SCPCs in patients with SCD. The clinical impact of a 100% inhibition of P-selectin was not fully elucidated before the trial initiation, and the applicant addressed this issue by defining AEs of special interest. Higher rates for AEs related to the hemostatic system were found in patients receiving HD crizanlizumab compared to placebo, but were mostly laboratory abnormalities without clinical translation. Updated safety data from the A2202 study revealed that SelG1 and SEG101 have comparable safety profiles. The remaining uncertainties, including long-term safety, will be addressed in the ongoing phase III A2301 study. Due to limited data on the use of crizanlizumab in pregnant women, healthcare professionals are encouraged to report all pregnancies through a Pregnancy Intensive Monitoring (PRIM) program.

The applicant applied for a conditional marketing authorization (CMA), which is intended for medicines: (1) addressing unmet medical needs; (2) aimed at treating, preventing or diagnosing seriously debilitating or life-threatening diseases; and (3) whose immediate availability outweighs the risks of not having comprehensive data. The CHMP agreed that SCD is a debilitating, life-threatening disease, still associated with a decreased life expectancy. Current therapy for SCD is preventive, limited to a few eligible patients, not well tolerated or accepted and not entirely effective, so the unmet medical need was also agreed. Moreover, crizanlizumab exhibits a new mode of action compared to existing therapies and offers an alternative or add-on therapeutic option.

Of note, CMAs are granted when it is likely that the MAH will provide comprehensive safety and efficacy data at a later stage. For that purpose, the MAH initiated in 2019 a multicenter, placebo-controlled phase III A2301 trial, whose primary analysis is planned for 2025. The design of this trial followed scientific advice given by the CHMP. Patients are being randomized 1:1:1 to crizanlizumab 5 mg/kg, 7.5 mg/kg, or placebo. In addition to confirming the efficacy and safety of crizanlizumab at the currently approved dose (5 mg/kg), the study will also assess whether a higher dose could further reduce the frequency of VOCs.

Crizanlizumab had a favorable safety profile that can be adequately managed via routine risk minimization measures. Therefore, making crizanlizumab available to patients while collecting comprehensive efficacy and safety data in the ongoing phase III study is not expected to represent a risk to public health.

## Conclusions

Based on the review of data on quality, safety, and efficacy, the EMA CHMP concluded by consensus that the risk–benefit balance of crizanlizumab was favorable for the prevention of recurrent vaso-occlusive crises in patients with SCD aged 16 years and older.

## Disclosures

The authors have no conflicts of interest to disclose.
